# Alpha-synuclein Immunization Strategies for Synucleinopathies in Clinical Studies: A Biological Perspective

**DOI:** 10.1007/s13311-022-01288-7

**Published:** 2022-09-09

**Authors:** Luisa Knecht, Jonas Folke, Richard Dodel, J. Alexander Ross, Alexandra Albus

**Affiliations:** 1 Chair of Geriatric Medicine, University Duisburg-Essen, Essen, Germany; 2 Centre for Neuroscience & Stereology, Department of Neurology, Bispebjerg-Frederiksberg Hospital, University Hospital of Copenhagen, 2400 Copenhagen, Denmark

**Keywords:** Alpha-synuclein, Parkinson’s disease, Passive immunization, Active immunization

## Abstract

The therapeutic strategies currently available for neurodegenerative diseases such as Parkinson’s disease target only the symptoms of the disease. Parkinson’s disease, dementia with Lewy bodies, and multiple system atrophy can be summarized as synucleinopathies, as they are all characterized by the aggregation and accumulation of alpha-synuclein (α-syn) in the brain. Targeting α-syn by its formation and progression opens a new and promising disease-modifying therapeutic strategy. Thus, several distinct immunotherapeutic approaches are currently being evaluated in clinical trials. The objective of this article is to review, from a biological perspective, the most important properties of these passive and active immunotherapies to point out their relevance and suitability for the treatment of synucleinopathies.

**Supplementary Information:**

The online version contains supplementary material available at 10.1007/s13311-022-01288-7.

## Introduction

Parkinson’s disease (PD), the second most common neurodegenerative disease after Alzheimer’s disease (AD), is histopathologically characterized by the accumulation of cytoplasmic proteins, specifically, the accumulation of neurofibrillary alpha-synuclein (α-syn) aggregates. α-syn pathology can be characteristically found in the substantia nigra [1, 2, 3], although its propagation is not restricted to this brain region alone. Distinctive pathogenic α-syn aggregates can be found throughout the whole brain, as well as in multiple peripheral organs such as the gut or skin [4, 5, 6, 7, 8, 9, 10, 11]. Apart from PD, neurofibrillary α-syn aggregates can be found in neurons of other neurodegenerative diseases such as dementia with Lewy bodies (DLB) as well as in cytoplasmic glial inclusions in oligodendrocytes in multiple system atrophy and peripheral neuronal α-syn deposits in pure autonomic failure (PAF), hence collectively called α-synucleinopathies [12]. The latter is characterized by general dysfunction of the autonomic nervous system (ANS) which presents with clinical symptoms without motor or cognitive impairment such as severe orthostatic hypotension (OH) as its cardinal clinical hallmark [13, 14, 15]. In addition to their main pathology, these neurodegenerative diseases have one thing in common: the lack of targeted treatments to effectively modify the disease course. Few available treatments focus on managing symptoms including levodopa which works through substitution of dopamine or deep brain stimulation to alleviate motor symptoms in PD [15]. Current treatments for PAF have comparably poor efficacy in halting disease progression, and the gold standard of (non-)pharmacological treatment of OH has exploited the possible treatment capacity [14]. As PAF patients have an increased risk of phenoconversion into other α-synucleinopathies [16, 17] with central involvement such as MSA, PD, and DLB by α-syn progression from the ANS ganglia and nerve fiber to the CNS, patients could highly benefit from α-syn immunization strategies to prevent pathology progression.

Under various conditions that are still not entirely understood, the physiological 14-kilodalton (kDa) [18], “natively unfolded” [19], intraneuronal protein undergoes conformational changes to assume a highly organized β-sheet structure that tends to accumulate into insoluble cytotoxic species [20]. Because it has been suggested that this protein plays a causative role in PD development and progression, it is considered an attractive target for the development of novel therapies to replace current treatments that only target the symptoms of the disease.

Schenk et al. first described a vaccination strategy for amyloid-β in AD [21], and Masliah et al. [22] pioneered such a strategy targeting α-syn in a mouse model of PD. The authors were able to observe positive effects of C-terminal-targeting antibodies on the degradation of α-syn aggregates after immunization with human α-syn [22, 23].

Because multiple passive and active strategies for immunization against α-syn are currently being investigated in clinical trials (Tables 1 and 2), this review aims to elucidate the current knowledge about and progress of this promising approach. This review specifically focuses on the biological characteristics of various lead compounds, aiming to identify individual and concordant antibody properties to evaluate and discuss the relevance and suitability of these compounds in immunization strategies for synucleinopathies.

**Table 1:** **Passive immunization against α-synuclein. Candidates for PD in clinical testing.** Summary of basic data of passive immunization strategies targeting α-synuclein currently enrolled in clinical testing. *n/a* information not available

**Name**	**Development**	**Phase (status) of** **clinical trial and** ***NCT number***	**Target (epitope)**	**Fab fragment** **(paratope)**	**Fc region** **(IgG** **subclass)**	**Glycosylation at** **Asn-297**	**Glycosylation at** **other** **glycosylation sites**	**Origin**	**References**
**ABBV-0805**/ BAN0805	AbbVie, BioArctic Neuroscience AB	Phase I in PD patients *NCT04127695* (withdrawn): recruitment stopped. Phase I trial ended due to strategic considerations	Preferably aggregated α-syn (ABBV-0805; 118-fold higher K_D_ against aggregated α-syn) and predicted within aa 121–127 (based on mAb47 antibody epitope mapping)	n/a	IgG1/4 with possible modification to reduce complement component 1q (C1q) binding (suggested by general strategy)	n/a	n/a	Humanized mAb47	[65, 74, 86, 107, 108, 109, 110]
**BIIB054**/ *cinpanemab*/ NI-202	Biogen Int Neuroscience GmbH, Neurimmune	1) Phase II in PD patients, SPARK study. *NCT03318523* (Terminated—did not meet its primary outcome) 2) Phase I in Japanese PD patients. *NCT03716570* (Terminated based on *NCT03318523*) 3) Phase I in healthy individuals and early PD patients *NCT02459886* (completed)	Aggregated, fibrillar, preferably oxidized α-syn at N-Terminal aa 4–10 (FMKGLSK)	**VH CDR1**: KAWMS/GFDFEKAWMS **VH CDR2**: RIKSTADGGTTSYAAPVEG **VH CDR3**: AH **VL CDR1**: SGEALPMQFAH **VL CDR2**: KDSERPSVL **VL CDR3**: QSPDSTNTYEV	IgG1	n/a	Incorporates glycosylated human IgG1 HC and lambda LC constant region sequences	Fully human, Isolated from B-cell library	[65, 66, 68, 77, 89, 109, 111, 112]
**Lu AF82422**	H. Lundbeck A/S, Genmab A/S	1) Phase II in MSA patients. NCT05104476 (recruiting) 2) Phase I in healthy individuals and PD patients *NCT03611569* (completed)	recognize all major species of α-syn (monomeric and oligomeric; N- or C-terminal truncated forms)	n/a	IgG1	n/a	n/a	Human	[76, 80, 82, 113]
**MEDI1341**/ TAK-341	Astra Zeneca, Takeda Pharmaceutical Company, MedImmune (originator)	1) Phase I in PD patients. NCT04449484 (completed) 2) Phase I in healthy individuals. *NCT03272165* (completed)	C-terminus of aggregated, monomeric α-syn; supposedly within aa 102–130 (suggested by preclinical data)	n/a	IgG1 with reduced effector function by triple mutation	n/a	n/a	Fully human; generated from lead isolate asyn0087, matured to aslo054 backbone	[75, 79, 85, 113, 114]
**PRX002**/ *prasinezumab*/ RO7046015/ RG7935/ NEOD002	Prothena Biosciences Limited, Hoffman-LaRoche	1) Phase IIb in early PD patients. PADOVA study, NCT04777331 (recruiting) 2) Phase II in PD patients. PASADENA study, *NCT03100149* (active, not recruiting) 3) Phase I in PD patients. *NCT02157714* (completed) 4) Phase I in healthy individuals. *NCT02095171* (completed)	Aggregated α-syn at C-terminal around aa 122, supposedly aa 118–126 (suggested by preclinical data of E94)	**VH CDR1**: SISSGGGSTYYPDNVKG **VH CDR1**: GGAGIDY **VL CDR1**: KSIQTLLYSSNQKNYLA **VL CDR2**: WASIRKS **VL CDR3**: QQYYSYPL	IgG1	n/a	n/a	Humanized version of murine 9E4 antibody	[58, 65, 69, 70, 71, 89, 109, 113, 115, 116, 117]

**Table 2:** **Active immunization against α-synuclein. Candidates for PD in clinical testing.** Summary of basic data of active immunization strategies targeting α-synuclein currently enrolled in clinical testing. n/a information not available

**Name/** **synonyms**	**Development**	**Phase (status) of clinical trial and** ***NCT number***	**Vaccination compounds**	**References**
**AFFITOPE**^**®**^ **PD01A**	AFFiRiS, Austria	Currently six registered trials: 1) Phase I in PD-GBA patients. *NCT02758730* (withdrawn) 2) Phase Ib in early PD patients. *NCT02618941* (AFF008AA, second dose for patients from AFF008, AFF008E, AFF008A) (safety trial: completed) 3) Phase Ib in early PD patients. *NCT02216188 (AFF008A), inclusions from prior studies AFF008, AFF008E* (completed) 4) Phase I in early MSA patients, *NCT02270489 (AFF009)* (completed) 5) Phase I, safety and tolerability in early PD patients. *NCT01568099 (AFF008)* (Completed) 6) Phase Ib, extension study, long-term effect, in PD patients. *NCT01885494* (AFF008E) (completed)	Immunogenic peptide mimicking the C-terminal human a-syn region (native epitope: DMPVDPDN) + immunological adjuvant aluminum hydroxide + Peptide carrier Keyhole limpet hemocyanin (KLH)	[64, 89, 93, 94, 95, 96, 97, 113, 116, 118]
**AFFITOPE**^**®**^ **PD03A**	AFFiRiS, Austria	1) Phase I in early PD patients *NCT02267434 (AFF011)* (completed) 2) Phase I in early MSA patients NCT02270489 (AFF009); (Completed)	n/a (mimicking sequence) + Immunological adjuvant aluminum hydroxide + KLH	[89, 93, 96, 97, 113, 119]
**UB-312**	United Biomedical, Inc. (United Neuroscience Ltd.)	Phase I in healthy individuals. *NCT04075318* (completed) Phase I Part A in healthy individuals, PART B in PD patients (H&Y ≤ III). *NCT04075318* (Active, not recruiting)	Heterologous T-helper cell epitope linked directly or through a heterologous spacer to a B-cell epitope; epitope: C-terminal region of α-syn within aa G 111–D 135 (suggested by preclinical data)	[99, 100, 101, 102]

## Biochemistry and Physiology of Alpha-synuclein

α-syn, synonymously termed precursor of the non-Abeta component of Alzheimer’s disease amyloid (NAC), is a 14-kDa, 140-amino acid [18], intraneuronal protein encoded by the *SNCA* gene, which is located on chromosome 4 [24]. α-syn is one of three members of the synuclein family, which includes two homologous proteins, namely beta (β)-synuclein [18] and gamma (γ)-synuclein [25].

**Fig. 1:**
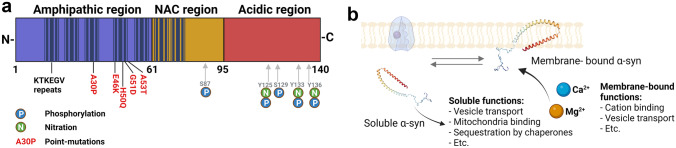
**Primary structure of α-syn: a**) The structure of α-syn can be divided into three distinct domains: the amphipathic N-terminal domain, associated with membrane binding, a middle NAC domain that has been suggested to play a key role in the protein’s aggregation properties, and a proline-rich acidic C-terminal. Most PD-associated missense mutations are located within the first 61 amino acid residues (in the blue region). The majority of posttranslational modifications are located in the acidic region (S: serine; P: phosphorylation site; Y: tyrosine; N: nitration site). **b**) α-syn is present as membrane-bound α-syn and soluble α-syn in equilibrium mediating different functions based on its localization, such as the cation-binding region on the free acidic region as a membrane bound and mitochondria binding in the cytoplasm. Modified from [105] and [106]

α-syn is referred to as “natively unfolded” because it does not have a defined three-dimensional structure in aqueous solution [19]. The primary structure of α-syn is mostly divided into three distinct domains to which different characteristics and functional roles have been attributed (Fig. 1A). The most interesting motif for PD research, comprising amino acid residues 61–95, is termed the NAC region because it was first identified in amyloid plaques of AD patients [26]. This highly hydrophobic center domain is believed to promote aggregation and amyloidogenic fibril maturation [26, 27, 28]. Confirming these findings, isolated NAC has been shown to form amyloid fibrils [29].

**Fig. 2:**
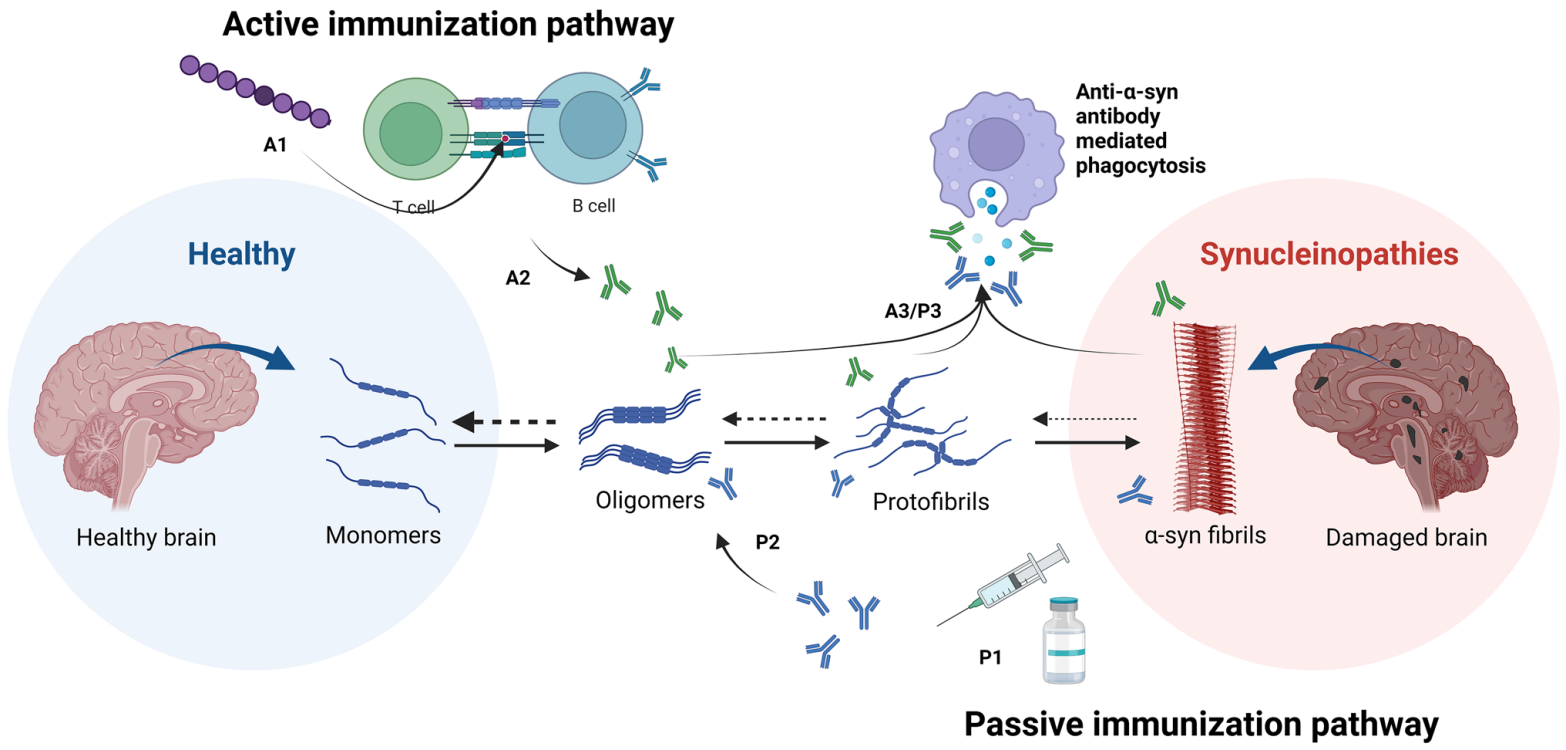
**Pathological pathway of α-synuclein aggregation**. Abnormal misfolded α-syn monomers form oligomeric structures with intrinsic properties to form proto-fibrils which turn into insoluble fibrils forming pathological inclusions, e.g., Lewy bodies in PD and DLB, and glial cytoplasmic inclusions in MSA. Immunization can potentially reverse α-syn accumulation and further reduce toxic effects. The active immunization pathway acts with immune presentation of a small fragment of α-syn (**A1**), and the immune system reacts to the peptide as foreign and produces antibodies by B cells in a T-cell-dependent manner (**A2**). The antibodies bind to the fragment alignment on α-syn and facilitate phagocytosis and protein clearance reduces the pathological effects (**A3**). Passive immunization circumvents the T-cell-dependent immune system by injection of in vitro-produced antibodies (**P1**) recognizing different structures of α-syn (**P2**) and mediates phagocytosis of α-syn (**P3**). Both strategies would be preferred in the early stages of α-syn accumulation, preventing the effects of abnormal toxic aggregation

The amphipathic lysine-rich amino terminus (residues 1–60) contains highly conserved hexameric imperfect motif (primary KTKE(G/Q)V) repeats [26] that modulate membrane interactions and thus were found to have alpha-helical properties [30, 31, 32]. Interestingly, all of the identified mutations associated with cases of familial PD and other synucleinopathies (e.g., A53T [33] and A30P [34]) were found to occur in these first 61 amino acid residues (Fig. 1).

The disordered proline-rich acidic carboxy-terminal (C-terminal) tail (residues 96–140) is the major site of posttranslational modification (PTM). This site regulates interactions with metals such as Ca^2+^, which is thought to increase binding to lipids [35] (Fig. 1B), small molecules, and proteins and to exhibit chaperone activity [36].

Although this protein is primarily located in the nucleus [37] and presynaptic terminals of neurons [18, 38], it has been reported to be present, albeit in much smaller amounts, in other cell populations, such as red blood cells [39] or enteroendocrine cells (EEC) [7]. The latter play a considerable role in the gut-to-brain axis hypothesis of PD development, which suggests an ascending α-syn pathology that originates in the gut and is transmitted from the enteric nervous system to the brain via the vagus nerve [40, 41].

The cellular functions of α-syn remain unknown. However, many aspects, such as its presynaptic localization [37, 38], its known interaction with SNARE proteins [42], which form a protein complex that mediates vesicle fusion, and the deficiency in synaptic transmission in cells lacking α-syn [43, 44], suggest its involvement in synaptic plasticity and synaptic vesicular transport regulation.

A major step in the pathogenesis of PD is the misfolding of α-syn monomers into oligomers or fibrils and their accumulation into cytotoxic aggregates [45]. During this process, α-syn undergoes conformational changes and transitions from its natively unfolded structure to a highly organized β-sheet structure (Fig. 2). While both conformations are generally linked to cytotoxicity, the current opinion shifts to the assumption that oligomers rather than fibrils exhibit this property [46]. This is due to low fibril cytotoxicity in cell culture experiments and inconsistent correlation of clinical symptoms to Lewy body formation and their α-syn isoform composition [46, 47, 48, 49, 50]. The oligomeric isoform varies in the possible structural conformation which includes spherical, chain- and pore-like, and tubular structures [51, 52, 53]. Consequently, oligomers have come into focus as therapy targets, although the research is hampered by their heterogenous nature [54].

In addition to mutations in the SNCA gene, including point mutations (e.g., A53T [33] and A30P [34]) or gene multiplication mutations [55, 56] in familial PD, posttranslational modification has been suggested to lead to aggregate formation; phosphorylation is a major mechanism of posttranslational modification in addition to truncation [57, 58] and ubiquitination [59]. α-syn that was recruited to synucleinopathic lesions was found to be phosphorylated at S129 [60].

It has been suggested that neurodegeneration may result from downstream microglia-mediated inflammatory responses. Aggregated α-syn, primarily in the form of fibrillar species [61], has been shown to activate microglia in the substantia nigra, thereby causing inflammatory processes that lead to dopaminergic neurodegeneration [62, 63, 64].

## Passive Immunization Candidates

As one of the principal approaches of current PD immunoregulatory therapies, passive immunization is based on the direct administration of antibodies targeting different domains of α-syn. Despite the required frequent administration of this type of therapy, the ability to control various parameters, including the binding affinities, the reliable regulation of antibody concentration, the ability to quickly intervene when side effects arise and the ability to bypass T-cell stimulations, makes passive immunization an advantageous treatment [65].

BIIB054/*cinpanemab*, a monoclonal antibody from Biogen, that binds almost exclusively to aggregated α-syn but also forms plasma complexes with α-syn [66], was tested for safety and efficacy in 357 patients in the randomized double-blinded SPARK study. Patients with early PD (≤ 3 years and Hoehn and Yahr stage (H&Y) ≤ 2.5; including a dopamine-transporter single-photon emission computed tomography (DaT-SPECT) consistent with degenerative Parkinsonism) were administered BIIB054/*cinpanemab* with a delayed start design for up to 72 weeks. Changes and improvements in the Movement Disorder Society-Unified Parkinson’s Disease Rating Scale (MDS-UPDRS) total score or MDS-UPDRS Part I, II, and III scores were considered the primary endpoints; DaT-SPECT changes from baseline to week 52 were chosen as secondary outcomes. The SPARK study did not meet its primary nor secondary endpoints, showing that BIIB054/*cinpanemab* treatment, indicated by measures of disease progression (MDS-UPDRS) and changes in DaT-SPECT imaging, did not differ from those of placebo over a 52-week period [67, 68]. ABBV-0805 from AbbVie, a humanized monoclonal antibody preferably binding aggregated forms of α-syn at the C-terminal, showed good tolerance and safety in phase I, leading to continuation in a disease group. During a placebo-controlled Phase Ib with 32 mild to moderate PD patients, the trial was withdrawn owing to strategic reasons.

Three additional candidates (Lu AF82422, MEDI1341, and PRX002/*prasinezumab*) are currently being evaluated for passive immunization against PD and/or MSA in phase I–III clinical trials (Table 1). PRX002/*prasinezumab* is the most advanced and entered phase II testing in June 2017 (NCT03100149). PRX002/*prasinezumab* showed good safety and tolerability in phase I studies in healthy and PD patients and reduced free serum α-syn in a dose-dependent manner [69, 70]. Currently, it is evaluated in the PASADENA study re-enrolling 316 early stage PD patients and is expected to be completed in 2026 [70]. Recently, the company announced a trend toward a beneficial effect on the clinical MDS-UPDRS part III motor scale on their secondary endpoint. However, the last update on the 52-week phase II trial did not reveal significant impact of treatment with PRX002/*prasinezumab* compared to placebo on primary outcome measures [71].

The MEDI1341 monoclonal antibody from Astra Zeneca has been investigated in parallel running two phase I studies in healthy individuals and PD patients, with no data disclosed thus far.

The last currently tested passive immunization candidate, Lu AF82422 from Lundbeck A/S, has recently launched a phase II study in MSA patients after completing phase I studies in healthy individuals and PD patients with no reported results.

These compounds differ to a certain extent in terms of their biological composition.

Based on the results of immunization experiments [22, 72] suggesting that antibodies that target the C-terminus are more effective in clearing α-syn, Prothena Biosciences Ltd. in collaboration with Hoffmann-LaRoche developed the C-terminus-targeting antibody PRX002/*prasinezumab*. This humanized (9E4 clone) monoclonal antibody binds to aggregated α-syn at approximately amino acids (aa) 122 [65], supposedly within aa 118–126, in accordance with preclinical testing with its parental murine molecule, 9E4 [73]. Generally, most passive immunization candidates presented herein follow the same approach: all the antibodies recognize rather short epitopes that are predominantly localized to the C-terminus of α-syn. Preclinical proof that this epitope region is targeted has been demonstrated for the murine version of ABBV-0805, namely mAb47, which binds an epitope at approximately aa 121–127 or 122–125 [74]. Other therapeutic antibodies were suggested to target either an epitope within aa 102–130 (MEDI1341, according to lead isolate asyn0087 [75]) or 112–117 [76] (AF 82,422), a region that H. Lundbeck A/S considers to be best for the treatment of synucleinopathies.

As truncation plays a major role in the pathological process of α-syn aggregation [57], the suggestion was raised that these antibodies might generally inhibit the formation of neurotoxic species by binding to the same sequence as the cleaving proteases [58].

Nevertheless, BIIB054/*cinpanemab*, which has critical epitope residue side chains within the first ten aa of the N-terminus [77], recently showed negative results in a large phase II study, whereas the large PASADENA study evaluating PRX002/*prasinezumab*, targeting the C-terminal of α-syn, showed positive results in their secondary endpoints that led to the launch of a phase IIb study (PADOVA). This discrepancy between large clinical trials highlights the cumbersome strategy of targeting α-syn and designing clinical trials. Several reasons could explain the different results. First, there are difference in binding sites on α-syn, and a specific epitope could be more advantageous to target. Second, the binding characteristics and effects of the individual antibodies could differ. Third, the composition of the cohorts could have a tremendous effect on positive trial outcomes, combined with more refined primary and secondary outcome measurements. These together with others, are all important considerations for evaluating successful clinical interventions.

Interestingly, no antibody that targets the middle domain of α-syn, carrying the non-amyloid-β component (NAC) region, even though its neurotoxic and aggregation properties are attributed to this region [78], has yet moved into clinical evaluation.

However, all antibodies have been shown to successfully recognize the main pathological structures of α-syn, albeit with different affinities. Although PRX002/*prasinezumab* [69], BIIB054/*cinpanemab* [77], MEDI1341 [79], ABBV-0805 [110], and Lu AF82422 [80] are documented to have higher affinities for aggregated, oligomeric, fibrillar or protofibrillar species, MEDI1341 (120-fold) and BIIB054/*cinpanemab* (800-fold) raised the hypothesis that the greatest clinical efficacy may be achieved with an antibody that also clears the monomeric α-syn “building blocks” [79]. Since the pathological progress in PD and other synucleinopathies such as MSA are not fully understood, accumulating evidence suggests that toxic cell-to-cell transmission of α-syn spreading is indeed an important factor of disease pathology. Thus, clearance of all extracellular α-syn species, monomeric and oligomeric, would not only block recipient cellular uptake but also attenuate spreading. To date, several preclinical studies have explored both clearance and blocking of α-syn species spreading in a variety of animal models with great success (reviewed by [81]). However, it is poorly understood which mechanisms are responsible for spreading, and if positive results from preclinical animal models can be translated into human trials. With that uncertainty in mind, prevention of cell-to-cell transmission, blocking of cellular uptake, and sequestration of α-syn to reduce oligomerization may be a robust immunotherapeutic strategy. This strategy seems also to be applied by Lundbeck A/S and their candidate, LU AF82422, which also targets all major species, mono- and oligomeric as well as N- and C-terminal truncated forms [82]. Although, both BIIB054 and *prasinezumab* are designed to preferably target aggregated forms of sequestration α-syn, they were also able to bind to monomeric α-syn in serum [66, 70].

A further relevant aspect of interest is the structure of the antibody, which determines its functional properties. Although recognition and neutralization by antigens are mediated by the antibody’s Fab fragment, phagocytosis and complement activation involve binding of the Fc fragment. Therefore, the IgG subclass and its glycosylation pattern prove to be of high importance.

IgG can be divided into four subclasses, namely IgG_1_, IgG_2_, IgG_3_, and IgG_4_, whereby IgG_1_ is the most abundant in human serum. Even though these subclasses share over 90% sequence homology, there are major differences in their binding and functional properties. These differences arise mostly due to variations in the hinge region, which is a flexible linker between the Fab regions and the Fc domain, and structural differences in the N-terminal Fc tail (formed by the CH_2_ and CH_3_ domains). As the hinge region defines the spatial arrangement of the Fab arms and Fc region in relation to each other, its flexibility and length determine the accessibility of the binding sites for innate receptors and adaptor molecules, including complement component 1q (C1q) and Fc gamma receptors (FcγR) on innate immune effector cells, such as phagocytes. Accordingly, IgG_3_, with a long flexible hinge region, is typically a potent trigger of effector mechanisms, as is IgG_1_. In contrast, the binding of the IgG subclasses IgG_4_ and IgG_2_ to most FcγR and C1q molecules is reduced, which, in addition to less flexible Fab arms (IgG_4_ > IgG_2_ [83]), appears to be caused by certain variations in the aa sequence [84].

Antibodies of the IgG_1_ subclass are the most common therapeutic candidates for PD. Interestingly, the antibody MEDI1341 was engineered by the introduction of triple mutations in the IgG_1_ Fc region to reduce its effector function to minimize the risks of bystander inflammatory responses [75, 85]. Similarly, BioArctic presented the general idea of protofibril-binding antibodies that are of either the IgG_1_- or IgG_4_-type with reduced complement activity (e.g., by combination of CH_3_ (IgG_1_) and CH_2_ (IgG_4_), changes in glycosylation, mutagenesis of asparagine 297 (Asn-297) or modification of the C1q binding site) [74, 86]. It is unclear whether ABBV-0805 is modified in any of these ways.

As previously mentioned, the glycosylation patterns of the Fab and Fc domains, and most importantly the distinct glycan composition, influence the effectiveness of a comprehensive spectrum of IgG-mediated functions, such as antibody-dependent cellular phagocytosis (ADCP), complement activation, antibody-dependent cell-mediated cytotoxicity (ADCC) or anti-inflammatory response induction [87]. One reason might be the open conformation of the Fc region upon the interaction of glycan structures with certain Fc amino acids that then allows easier binding to molecules or receptors. In addition, interactions among glycan structures can increase the binding of IgG to its associated FcγR. In that regard, the conserved glycosylation site at Asn-297 in the CH_2_ domain is suggested to play a crucial role because it influences the quaternary structure of the Fc region [84, 87].

Hence, it seems to be of high importance to assess the oligosaccharide composition and structures of antibodies in the course of preclinical experiments. Moreover, these findings open up the possibility of glycoengineering, allowing for the optimization of an antibody’s therapeutic functions.

Nevertheless, to date only a few companies have addressed this major characteristic of their lead antibodies. For example, Biogen Int. Neuroscience GmbH engineered an antibody (BIIB054/*cinpanemab*) that included a glycosylated human IgG_1_ heavy chain and lambda light chain constant region sequence without providing further information about the exact glycosylation sites, compositions, or purpose [77]. However, the possibility of glycoengineering and its results are found in multiple patent documents about companies’ preclinical examinations of multiple antibody variations, although these results cannot be clearly associated with the therapeutic antibodies presented here. BioArctic Neuroscience AB, for instance, addresses the possibility of altering oligosaccharide structures to reduce C1q binding and thus minimize the risk of proinflammatory processes [74]. Most companies documented glycosylation sites in amyloid-beta-targeting antibodies but did not provide data on the glycan composition or on its intended effect (Table 1). There was no particular mention of glycosylation at Asn-297, even though this site is generally considered the most important glycosylation site in antibodies. It seems that while the benefits of glycosylation are widely known, they are not always fully investigated for lead antibodies, or the information is not publicly available.

Another antibody property to examine is the origin. In addition to several investigations on this method, similar to our group, Biogen Int. Neuroscience GmbH invested in this approach. Both aducanumab (AD) and BIIB054/*cinpanemab* (PD) are products of similar technology platforms that yield antibodies from human memory B-cell libraries that most closely resemble naturally occurring antibodies [88, 89]. Although MEDI1341 [79] and Lu AF82422 [90] are also of human origin, ABBV-0805 and PRX002/*prasinezumab* are humanized versions of murine parent antibodies [65].

## Active Immunization Candidates

The second principle approach pursued in immunoregulatory therapy is active immunization. Vaccination uses the individual’s own immune system to elicit a specific immune response after the administration of an antigen. This treatment has multiple advantages over passive immunization, including less frequent administration and lower production costs [64]. The drawbacks of this approach include that vaccination does not allow the regulation of the strength of the immune response, and there is no guarantee of an efficient immunogenic reaction [64], especially in elderly people with diminished humoral and cellular responses (due to immunosenescence) [91]. When side effects occur, such as in the immunization trial of AN1792 in patients with AD, limiting the immune reaction may be difficult [92].

Currently, the safety, tolerability, and immunogenicity characteristics are being assessed for three hit molecules in phase I studies: Affitope PD01A and Affitope PD03A by AFFiRiS in PD and MSA and UB-312 in PD by United Biomedical Inc., United Neuroscience Ltd. (Table 2).

One critical issue related to the development of vaccines that target self-proteins, such as α-syn, is the generation of an antigen construct that balances the desired and unwanted immune responses. On the one hand, the antigen should serve as a B-cell antigen to stimulate site-specific antibody responses, but on the other hand, and of high importance, the antigen should not activate T cells to prevent autoimmunity resulting from inflammatory processes. Such side effects were observed in earlier AD vaccination studies, and these side effects resulted in the occurrence of meningoencephalitis [92].

The reported candidates Affitope PD01A/PD03A and UB-312 were specifically designed to avoid these side effects. Both Affitope PD01A and PD03A were developed by applying AFFITOME® technology [93]. This strategy uses so-called AFFITOPES, short peptides that mimic α-syn epitopes (mimotopes), as antigenic compounds. In contrast to the native α-syn amino acid sequence, these structures are foreign to the human immune system. Due to their length of approximately eight amino acids, mimotopes are short enough to not activate T cells but long enough to be presented in the context of the major histocompatibility complexes of antigen-presenting cells [93, 94].

In the case of Affitope PD01A, the peptide mimics the native C-terminal epitope DMPVDPDN [95, 96], which is a potent antigenic epitope capable of activating T-helper cells; this is a crucial step in inducing IgG responses. Indeed, the administration of PD01A resulted in the appearance of (low levels of) IgG antibodies that reacted with both PD01A and the targeted α-syn peptide sequence [95]. No preclinical data document the exact antigenic epitope of PD03A.

Furthermore, both vaccines include a keyhole limpet hemocyanin (KLH) carrier and are administered with aluminum hydroxide as an immunological adjuvant [64]. Because the KLH carrier is foreign to the immune system, it also functions as a potent activator of the B-cell response [95]. Phase I trials evaluated safety and tolerability of PD01A and PD03A in PD and MSA patients and both active treatments induced a sustained IgG antibody response against the immunization peptides [97], although PD03A induced significantly lower seroconversion in MSA and PD patients [97, 98]. In the short term the PD01A and PD03A showed no clinical improvements. After these two trials, the active immunization candidate PD01A was preferably used in phase Ib extension and phase II studies to evaluate clinical outcomes. In July 2021 AC Immune announced a phase II trial initiation of a new optimized PD01A formula, called AC-7104 [99].

The United Biomedical, Inc. and United Neuroscience Ltd. compounds UB-312 in PD, the related Alzheimer’s vaccine UB-311 [98], and active immunotherapies for multiple other diseases [99] were developed on the basis of the so-called UBITh® technique. Vaccines developed according to this technology are composed of heterologous (amino acid sequences that are not part of or are homologous to the wild-type sequence) T-helper cell epitopes that are linked either directly or indirectly through a heterologous spacer to a short B-cell epitope as an antigenic target site. The artificial T-helper cell determinant leads to an increased immune response that produces a high level of antibodies directed against the target, but the antigen itself is preferably immunosilent. Regarding the side effects of vaccination, the short peptide immunogen construct proved to not generate significant inflammatory responses [100].

As with PD03A, little detailed information about the B-cell epitope of the target antigenic site of UB-312 is available. However, patent literature reveals that the B-cell epitopes used for α-syn vaccination compounds should comprise a C-terminal fragment of ten to 25 amino acids, corresponding to the amino acid residues from glycine (G) 111 to aspartic acid (D) 135, or fragments thereof [101]. Very recently, the results of a safety and tolerability phase I trial 50 eligible healthy individuals were published. UB-312 was generally safe and well-tolerated and showed a positive seroconversion of 91.3% in all participants, which was highest in the high-dose recipients, and in CSF titers [102]. A phase Ib study in PD patients is currently planned.

## Outlook

A considerable number of immunization approaches have been initiated to reduce the sequelae of α-syn aggregation properties. Currently, no approach has entered a clinical phase III protocol. A large number of articles, especially in AD, have addressed the reasons and background of the lack of efficacy of current therapeutic options [103]. These include (1) the lack of preclinical models that fully mirror human clinical progression, (2) the administration of drugs to the wrong study population (e.g., too advanced disease stage), (3) an inappropriate study design, which does not consider the disease duration and development, (4) durability of the aggregation hypothesis for neurodegenerative disease, (5) addressing the adequate disease pathways, and (6) complexity of neurodegenerative diseases which cannot be addressed with a single treatment but needs multiple therapies.

Several challenges emerge when considering immunotherapy in synucleinopathies, such as intracellular α-syn aggregation, poor blood–brain barrier permeability of antibodies, and passively administered antibodies and antibodies endogenously produced after active vaccination. Currently, only IgG antibodies are being tested in passive immunization, and IgG_1_ has been the most promising to date, being the most common among treatment candidates for both AD and PD. A possible strategy to circumvent the accessibility of less permeable areas, could be to reduce the molecular weight of the antibodies. To overcome this, intrabodies or nanobodies should be considered as a possibility for future advancements. In that sense, translation discovery of antibodies should include the V(D)J information including the CDR regions which provide the opportunity to use the IgG subclass and other parameters as bases for choosing an antibody. 

As highlighted and documented for some therapeutic α-syn-targeting antibodies (Table 1), it may be appropriate to place more emphasis on glycoengineering as part of the drug development process. The targeted selection of the composition of N-glycans and the general glycosylation pattern of the Fab arms and Fc region would provide the possibility to further enhance and define certain functional aspects (e.g., ADCP or ADCC [87]) of antibodies as well as minimize their side effects, including inflammatory responses.

The potential negative outcome from clinical trials could also be appropriate for patient cohort composition and selection. One factor could be the specific stage of disease or a disease subtype, as previously discussed [81]. Furthermore, a combination of therapies could have the desired effect. Hence, a multidisciplinary approach with a combination of disease-modifying therapy drugs combined with a very specific patient selection process may be the future of curable strategies in synucleinopathies. In addition to α-syn-targeting antibodies, possible interactions between α-syn and tau implicate tau as a new potential therapeutic target for immunotherapy in PD [104].

## Supplementary Information

Below is the link to the electronic supplementary material.

Supplementary file1 (PDF 464 kb)

Supplementary file2 (PDF 508 kb)

Supplementary file3 (PDF 972 kb)

Supplementary file4 (PDF 1056 kb)

Supplementary file5 (PDF 524 kb)
